# Identification and validation of a gap junction protein related signature for predicting the prognosis of renal clear cell carcinoma

**DOI:** 10.3389/fonc.2024.1354049

**Published:** 2024-02-22

**Authors:** Yongsheng Huang, Wenyi Guo, Yuan Zeng, Xinrong Wang, Bohao Fan, Ying Zhang, Lei Yan, Gangli Gu, Zhao Liu

**Affiliations:** ^1^Department of Urology, Qilu Hospital, Cheeloo College of Medicine, Shandong University, Jinan, China; ^2^Department of Pancreatic Surgery, General Surgery, Qilu Hospital, Cheeloo College of Medicine, Shandong University, Jinan, China; ^3^Department of Anatomy and Neurobiology, Shandong Provincial Key Laboratory of Mental Disorders, School of Basic Medical Sciences and Qilu Hospital, Cheeloo College of Medicine, Shandong University, Jinan, China; ^4^Department of Surgery, Qihe County Traditional Chinese Medicine Hospital, Dezhou, China

**Keywords:** gap junction protein, clear cell renal cell carcinoma, biomarkers, prognostic model, cellular verification

## Abstract

**Background:**

Gap junction proteins (GJPs) are a class of channel proteins that are closely related to cell communication and tumor development. The objective of this study was to screen out GJPs related prognostic signatures (GRPS) associated with clear cell renal cell carcinoma (ccRCC).

**Materials and Methods:**

GJPs microarray data for ccRCC patients were obtained from The Gene Expression Omnibus (GEO) database, along with RNA sequencing data for tumor and paired normal tissues from The Cancer Genome Atlas (TCGA) database. In the TCGA database, least absolute shrinkage and selection Operator (LASSO) and Cox regression models were used to identify GJPs with independent prognostic effects as GRPS in ccRCC patients. According to the GRPS expression and regression coefficient from the multivariate Cox regression model, the risk score (RS) of each ccRCC patient was calculated, to construct the RS prognostic model to predict survival. Overall survival (OS) and progression-free survival (PFS) analyses; gene pan-cancer analysis; single gene survival analysis; gene joint effect analysis; functional enrichment analysis; tumor microenvironment (TME) analysis; tumor mutational burden (TMB) analysis; and drug sensitivity analysis were used to explore the biological function, mechanism of action and clinical significance of GRPS in ccRCC. Further verification of the genetic signature was performed with data from the GEO database. Finally, the cytofunctional experiments were used to verify the biological significance of GRPS associated GJPs in ccRCC cell lines.

**Results:**

GJA5 and GJB1, which are GRPS markers of ccRCC patients, were identified through LASSO and Cox regression models. Low expression of GJA5 and GJB1 is associated with poor patient prognosis. Patients with high-RS had significantly shorter OS and PFS than patients with low-RS (*p*< 0.001). The risk of death for individuals with high-RS was 1.695 times greater than that for those with low-RS (HR = 1.695, 95%CI= 1.439-1.996, *p*< 0.001). Receiver Operating Characteristic (ROC) curve showed the great predictive power of the RS prognostic model for the survival rate of patients. The area under curve (AUC) values for predicting 1-year, 3-year and 5-year survival rates were 0.740, 0.781 and 0.771, respectively. The clinical column chart was also reliable for predicting the survival rate of patients, with AUC values of 0.859, 0.846 and 0.796 for predicting 1-year, 3-year and 5-year survival, respectively. The GRPS was associated with immune cell infiltration, the TME, the TMB, and sensitivity to chemotherapy drugs. Further *in vitro* experiments showed that knockdown of GJA5 or GJB1 could promote the proliferation, migration and epithelial-mesenchymal transition (EMT) and inhibit apoptosis of ccRCC cells.

**Conclusion:**

GJA5 and GJB1 could be potential biological markers for predicting survival in patients with ccRCC.

## 1 Introduction

Among all types of cancer, renal cancer is the 16th most common cancer, accounting for approximately 1.8% of cancer-related deaths (1). Clear cell renal cell carcinoma (ccRCC) is the most common histological type of cancer and represents 75% of all cases (2, 3). More than 50% of renal cancer cases are detected through health examination (4). At the time of initial diagnosis, approximately 30% of patients have already developed distant metastasis (5–7). The 5-year survival rate for patients with ccRCC varies depending on the stage of the disease. The survival rate ranges from 20% to 95% for patients in the early, middle, and advanced stages, while for patients with metastatic ccRCC, the survival rate is only between 0% and 10% (8, 9). Early diagnosis plays a crucial role in improving overall survival (OS) and progression-free survival (PFS) in ccRCC patients. Therefore, it is clinically important to explore valuable prognostic indicators that can be used for personalized prognosis assessment and treatment planning, to ultimately improve the overall prognosis of patients.

Gap junction proteins (GJPs) are a family of hexamer-structured channel proteins that facilitate molecular and ion exchange between neighboring cells, thereby regulating various biological processes (10–12). The functional diversity of GJPs is attributed to variations in the molecular weight of the constituent proteins that form the gap junction channels (13). These proteins are involved in multiple biological processes, such as apoptosis, proliferation, immune response, and digestion (14, 15). Presently, 35 genetic diseases in humans are known to result from mutations in 11 different GJPs (16, 17). In addition, the obstruction of gap junction channels diminishes communication between immune cells and reduces the permeability of chemotherapy drugs. These findings suggest that GJPs potentially play a crucial role in cancer development and treatment, as well as in maintaining intercellular signal transmission and the stability of the tumor microenvironment (TME). To our knowledge, no reports have explored the clinical significance of GJPs in ccRCC, necessitating further investigation. This study identified a GJP-related prognostic signatures (GRPS) in ccRCC. Subsequently, an in-depth analysis was conducted to explore the clinical value of the GRPS in assessing ccRCC prognosis, as well as its influence on the tumor mutational burden (TMB) and the tumor microenvironment (TME). Furthermore, additional cytological experiments validated the analysis results, providing evidence that the GRPS may serve as a novel biomarker for predicting the survival prognosis of ccRCC patients.

## 2 Materials and methods

### 2.1 Bioinformatics analysis

**Figure 1:**
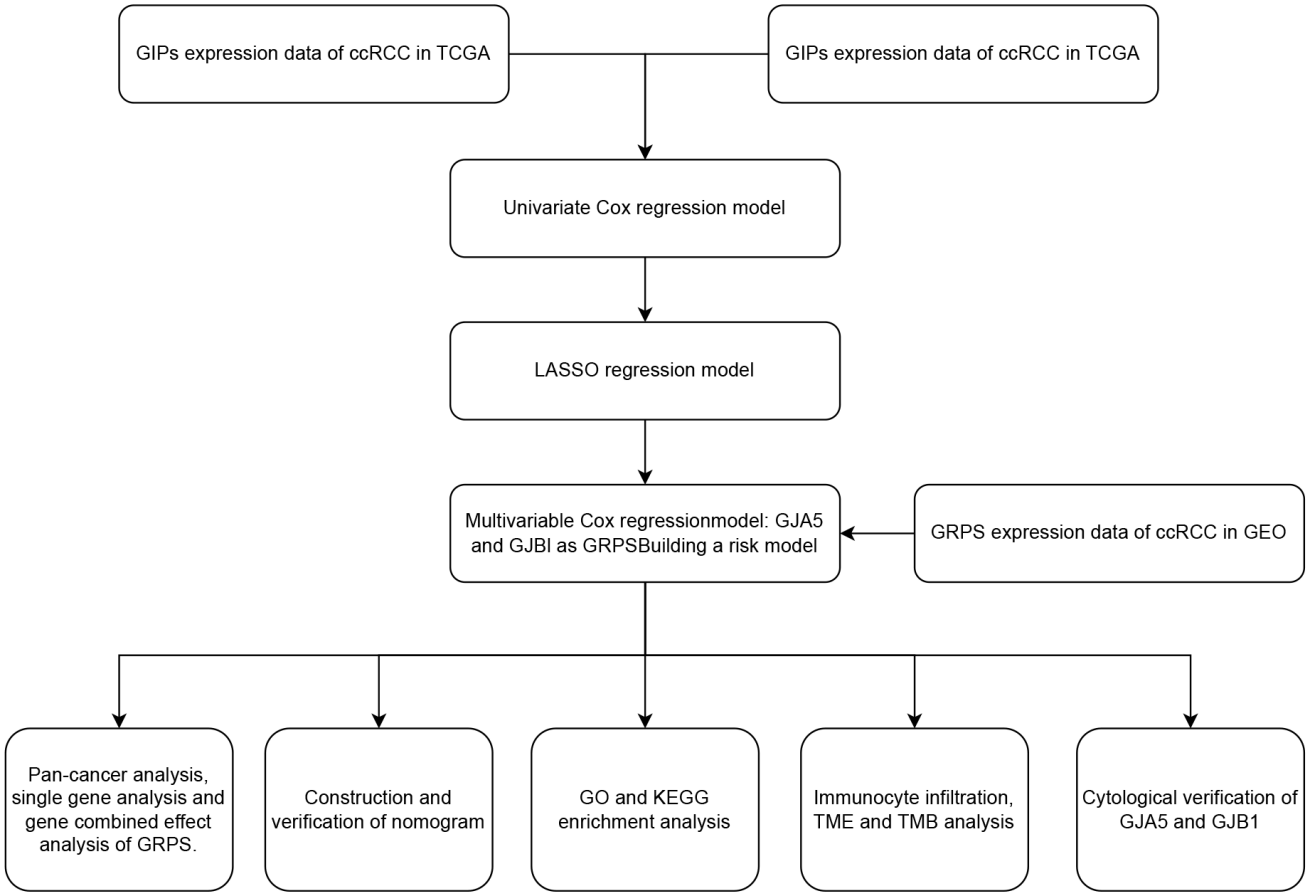
Workflow chart.

The RNA sequencing data, clinical trait data, pan-cancer data and simple nucleotide variation data of ccRCC were obtained from The Cancer Genome Atlas (TCGA) database (https://portal.gdc.cancer.gov/); Microarray data (GSE29609, GSE95425, GSE73731) were retrieved from Gene Expression Omnibus (GEO) database (https://www.ncbi.nlm.nih.gov/geo/). This study constructed and validated the GRPS for ccRCC prognosis using various statistical methods. The detailed workflow of this analysis is depicted in **Figure 1**. The general clinical information of the ccRCC patients in the TCGA database can be found in **Supplementary Table 1**.

#### 2.1.1 Identification of GRPS in ccRCC

A total of 21 GJPs were observed to be expressed in ccRCC. This study combines the survival information of ccRCC patients in the TCGA database (including survival status and survival time) with the gene expression data of GJP to generate a total sample (total sample, TS) for data analysis. Subsequently, a sequence of univariate Cox regression models (*p*< 0.001, KMP = 1 for filtration criteria), LASSO regression models, and multivariate Cox regression models was constructed for TS.

#### 2.1.2 Analysis of the combined effects of GJA5 and GJB1 on single gene and dual genes

Pan-cancer data for 33 tumors were obtained from the TCGA database. The differential expression of GJA5 and GJB1 in 33 cancers was examined via pan-cancer analysis. The difference analysis data of GRPS in ccRCC tumor tissue and normal tissue were obtained from TCGA database and GEO database (GSE73731 and GSE95425). Single-and dual-gene combined survival (Kaplan-Meier, [KM]) analyses were used to determine the significance of GJA5 and GJB1 in survival and prognosis of patients with ccRCC.

#### 2.1.3 Construction and validation of the prognostic risk score model

To further explore the prognostic significance of the GRPS in ccRCC, a computation was performed based on the coefficients derived from the multivariate Cox regression model, along with the corresponding gene expression levels. Subsequently, an risk scores (RS) prognostic model was constructed utilizing the RS values. The RS formula employed in the RS model was as follows:


Risk score = ∑(Coefficient of gene * expression of gene).

The high-RS group and low-RS group were divided according to the median RS. The RS prognostic model was developed and validated. The 70% of the samples randomly selected from the TS were assigned to the training dataset, while the remaining 30% of the samples and the GSE29609 dataset were used as the testing set (18), to confirm the generalizability of the GRPS. KM analysis, ROC curve analysis, and comparisons of clinical traits (including age, sex, grade, stage, and metastasis) between risk groups were also performed to assess the clinical significance of the GRPS.

#### 2.1.4 Construction of a clinical nomogram

This study developed a clinical nomogram incorporating patient clinicopathologic characteristics, risk groupings, and GJA5 and GJB1 expressions. Furthermore, calibration curve, ROC curve, and Decision Curve Analysis (DCA) analyses were performed to assess the clinical availability of the nomogram.

#### 2.1.5 Enrichment analysis

The biological function and underlying mechanisms of GRPS were investigated through gene enrichment analysis. Genes with statistical significance in the risk grouping (selected using the R package “Limma” with the criteria of *p*< 0.05, logFC = 1, and FDR< 1) were subjected to GO and KEGG enrichment analysis.

#### 2.1.6 Analysis of immune cell infiltration, TME and TMB

Alterations in the TME and the infiltration of immune cells have garnered significant attention in the field of cancer therapy. The mechanism triggering immune escape was investigated by examining the TME and immune cell infiltration in risk groups.

After the calculation of the levels of 22 immune cells and the abundance of TME-related molecules in patients with ccRCC, the differences in the levels of immune cells and the abundance of TME-related molecules between risk groups were analyzed, and *p*< 0.05 was considered statistically significant. TMB refers to the number of gene mutation sites that occur in tumor tissues and includes somatic gene coding errors, base substitutions, insertions or deletions (19). The TMB values of patients in the TS cohort were calculated based on simple nucleotide variation data from ccRCC patients in the TCGA database. The estimated TMB was calculated as the ratio of the total mutation frequency to the length of human exons (20). Finally, the TMB values of the ‘high-RS and low-RS groups were compared for any significant difference.

#### 2.1.7 Drug sensitivity analysis

The GDSC2 dataset used for drug sensitivity analysis was derived from the GDSC database (https://www.cancerrxgene.org). We calculated the susceptibility of ccRCC patients to 198 chemotherapeutic agents from the TCGA database. To provide accurate individualized treatment plans for patients, we used *p*< 0.001 as the filtering standard to analyze the difference in the sensitivity of patients with advanced ccRCC to targeted drugs according to risk group, which is helpful for providing medication guidance for different groups of patients with ccRCC.

#### 2.1.8 Statistical analysis

The analysis of the data and the generation of graphs were carried out with R language (version 4.2.1). The LASSO regression model, univariate and multivariate Cox regression models were constructed through the “glmnet” package; The “survival, survminer” package was used to perform KM analysis of the combined effect of a single gene and dual genes. The “timeROC” package was used to perform ROC curve analysis; The clinical nomogram was developed through the “survival”, “regplot” and “rms” packages; The “ggDCA” package was used for DCA analysis; GO enrichment analysis was carried out through the package “clusterProfiler, org.Hs.eg.db, enrichplot, ggplot2”; The “clusterProfiler, org.Hs.eg.db, enrichplot, ggplot2, circlize, RColorBrewer, ComplexHeatmap” package was used for KEGG enrichment analysis. We calculated the proportions of 22 immune cells in ccRCC tissue using the CIBERSORT algorithm and evaluated the abundance of related molecules in the TME using the R package “estimate”. The oncoPredict package was used to calculate a sensitivity score for ccRCC patients to chemotherapy drugs. The chi-square test was used to analyze the difference in clinicopathological information between the training set and the testing set. Unless otherwise stated in this paper, *p*< 0.05 was considered to indicate statistically significant.

### 2.2 Cell function experiment

#### 2.2.1 Cell culture and transfection

The HK2 and A498 cells were cultured in Dulbecco’s modified eagle medium (DMEM) supplemented with 10% fetal bovine serum (FBS) at 37°C in a 5% CO2 incubator; the 786-O cells were cultured in Roswell Park Memorial Institute-1640 (RPMI-1640) supplemented with 10% FBS at 37°C in a 5% CO2 incubator. Cells in logarithmic phase were chosen for functional experiments. Interference of GJA5 was performed using small interfering RNA (si-GJA5-NC; si-GJA5-1, si-GJA5-2, and si-GJA5-3) sequences synthesized by RiboBio Biotechnology (Guangzhou, China). The sequences used were as follows: si-GJA5-1 (AGGCTGATTTCCGGTGTGA), si-GJA5-2 (CATGGCTATCATAGTGACA), si-GJA5-3 (AATCCCTTCAGCAATAATA). Interference of GJB1 was performed using small interfering RNA (si-GJB1-NC; si-GJB1-1, si-GJB1-2, and si-GJB1-3) sequences synthesized by RiboBio Biotechnology (Guangzhou, China). The sequences used were as follows: si-GJB1-1 (GCTGCAACAGCGTTTGCTA), si-GJB1-2 (TGTTCCGGCTGTTGTTTGA), si-GJB1-3 (CGTGAACCGGCATTCTACT). Transfection was performed using Lipofectamine 3000 (Invitrogen, Shanghai, China). The relevant experiments were conducted 48 hours post transfection.

#### 2.2.2 The quantitative reverse transcription-polymerase chain reaction

Total RNA was extracted from HK2, A498, and 786O cells using TRIzol Reagent (Servicebio, Wuhan, China). The quality of the extracted RNA was assessed by measuring the OD ratio (A260/A280) using a Nano-400A spectrophotometer (Allsheng, Hangzhou, China). Subsequently, total RNA was transcribed into complementary DNA (cDNA) using the HiScript II Q RT SuperMix Kit (Vazyme). Amplified products were then detected using SYBR Green (Vazyme, Nanjing, China). β-actin was selected as the endogenous references. The specific primers used were GJA5: 5′-GAACACAGACAGGCAGAGGAT-3′ (F), 5′-GGAAGCTCAATCGCCCATC-3′ (R); GJB1: 5′-CCTGCACAGACATGAGACCA-3′ (F), 5′-AGAGCCATACTCGGCCAATG-3′ (R); and β-actin: 5′- CCTAGAAGCATTTGCGGT -3′ (F), 5′- GAGCTACGAGCTGCCTGACG-3′ (R).

#### 2.2.3 5-Ethynyl-2-deoxyuridine assay

An EdU Kit (Beyotime, Shanghai, China) was used to detect cell proliferation. Then, the A498 and 786O cells were incubated with EdU solution, fixed with 4% paraformaldehyde and infiltrated with Triton X-100 solution (Solarbio, Beijing, China). Then, the cells were stained with 4’,6-diamidino-2-phenylindole (DAPI; Beyotime, Shanghai, China). Ultimately, the EdU-positive cells (EdU+ DAPI-stained cells) were counted under a fluorescence microscope.

#### 2.2.4 Wound healing assay

The cells were seeded in 6-well plates. The confluent cell cultures were then scratched using a sterile tip. The wound healing process was monitored at different time points, and images of the scratches were captured using an inverted microscope after 12 hours.

#### 2.2.5 Transwell assay

Cell migration was assessed using the transwell assay. In this assay, we seeded 1.5×10^4^ cells into the upper chamber and cultured them in a serum-free medium, while the lower chamber was supplemented with DMEM or RPMI-1640 containing 10% FBS as a chemoattractant. After 24 hours of incubation, the cells that had migrated through the pores of the transwell membrane were fixed with 4% methanol. Subsequently, the sections were stained with crystal violet, images were captured and cell counts were performed using an inverted microscope.

#### 2.2.6 Western blot

Total protein was extracted using radioimmunoprecipitation assay (RIPA) lysis buffer (Servicebio, Wuhan, China) supplemented with phenylmethylsulfonyl fluoride (Beyotime, Shanghai, China) at a ratio of 50:1. The protein concentration was determined using the bicinchoninic acid (BCA) assay (Solarbio, Beijing, China). Subsequently, the samples were mixed with loading buffer and boiled for 10 minutes to denature the proteins before further analysis. Subsequently, 20 μg of protein was loaded into each lane and separated by sodium dodecyl sulfate-polyacrylamide gel electrophoresis (SDS-PAGE) with an 8-15% acrylamide gradient gel. The separated proteins were then transferred onto polyvinylidene fluoride (PVDF) membranes. To prevent nonspecific binding, the membranes were blocked with 5% nonfat dry milk in Tris-buffered saline supplemented with 0.1% Tween 20 (TBST) for a period of 2 hours. Primary antibodies against GJA5 (1:1,000; ABclonal, Wuhan, China), GJB1 (1:1000; ABclonal, Wuhan, China), GADPH (1:10,000; Proteintech, Wuhan, China), E-cad (1:1000; ABclonal, Wuhan, China), N-cad (1:1000; ABclonal, Wuhan, China), VIM (1:1000; ABclonal, Wuhan, China), Bax (1:1000; ABclonal, Wuhan, China), and Bcl-2 (1:1000; ABclonal, Wuhan, China) were incubated with the membrane overnight at 4°C. After the membranes were washed with TBST for 10 minutes, they were incubated with secondary antibodies (1:5000; Proteintech, Wuhan, China) for 2 hours, after which the membranes were washed 3 times with TBST. Finally, electrochemiluminescence (ECL, Thermo, China) was applied to visualize the results.

#### 2.2.7 Flow cytometric analysis

Apoptosis was detected with an annexin V apoptosis kit (Vazyme, Jiangsu, China). Fluorescence-activated cell sorting (FACS) was performed on a BD Accuri® C6 Plus [Becton, Dickinson, and Co. (BD) Biosciences, Franklin Lakes, NJ, USA] and analyzed by FlowJo software (https://www.flowjo.com/). Briefly, 1×10^6^ cells were collected and resuspended after adding 100 μL of binding buffer. Next, 100 μL of binding buffer containing 4 μL of annexin V-FIFC and 4 μL of propidium iodide (PI) staining solution were added. Finally, 400 μL of binding buffer was added to the culture tube, which was subsequently analyzed after 10 to 15 minutes using flow cytometry.

#### 2.2.8 Statistical analysis

All the statistical analyses were conducted using GraphPad Prism 8.0 software. Significance was determined using Student’s t-test or one-way analysis of variance (ANOVA), as appropriate. Each experiment was performed in triplicate, and *p<* 0.05 was considered to indicate statistical significance.

## 3 Results

### 3.1 Bioinformatics analysis results

#### 3.1.1 Identification of GRPS in the TCGA database

**Figure 2:**
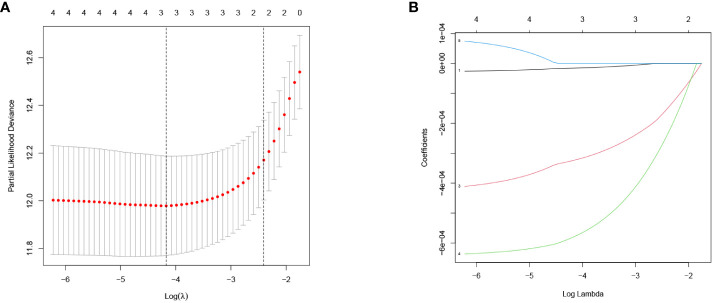
LASSO regression model of OR-GJPs: **(A)** Coefficient distribution diagram. **(B)** Parameter change diagram.

Firstly, a univariate Cox regression model for TS was constructed in this study, yielding five OS-related GJPs (OR-GJPs) that were most significantly related to the patients’ OS. To prevent data overfitting, a LASSO regression model for OR-GJPs was constructed (**Figures 2A, B**). Ultimately, a multivariate Cox regression model was constructed based on the outcomes of the LSAAO regression analysis. This model identified two genes (Gene GJA5 and Gene GJB1) with independent prognostic impacts on ccRCC (**Table 1**), which were identified as GRPS genes for subsequent data examination, clinical validation, and cellular functional experiments. The univariate Cox regression analysis for all 21 GJPs and the corresponding KM survival analysis for 5 GJPs with differential survival significance can be found in **Supplementary Table 2** and **Supplementary Figure 1**.

**Table 1:** Univariate and multivariate Cox regression models of GRPS.

	Univariate Cox regression model			Multivariate Cox regression model			
	HR	95% CI	*p*	coef	HR	95% CI	*p*
GJA5	0.9995	0.9993~0.9996	**<0.001**	-0.0005	0.9995	0.9993~0.9997	**<0.001**
GJB1	0.9992	0.9989~0.9994	**<0.001**	-0.0004	0.9996	0.9993~0.9999	**0.014**

The results with bold font indicate statistical significance (*p* < 0.05).

#### 3.1.2 Pan-cancer analysis, single gene analysis and combined effect analysis of GJA5 and GJB1

**Figure 3:**
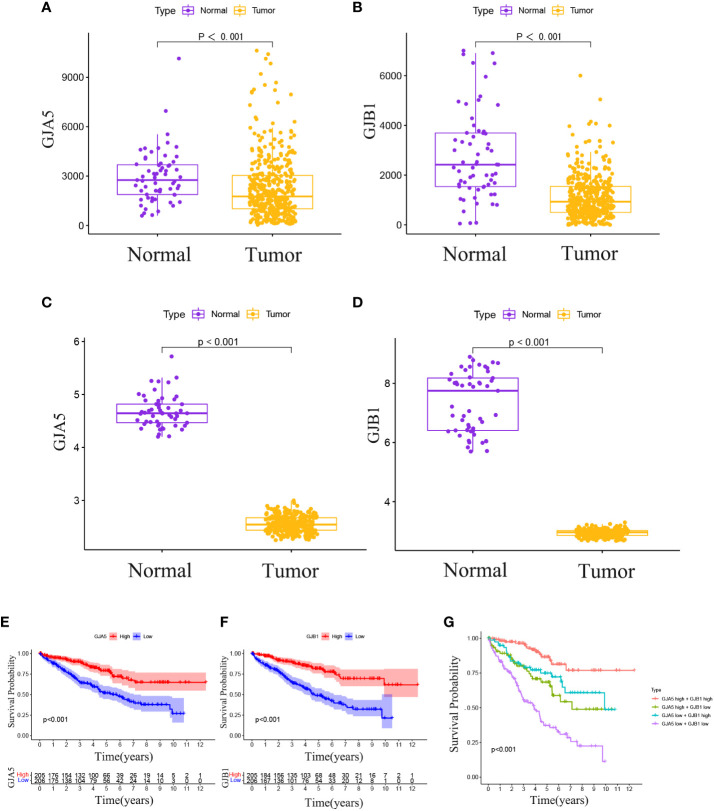
**(A)** In the TCGA database, differential expression of GJA5 in tumor tissues and paired normal tissues. **(B)** In the TCGA database, differential expression of GJB1 in tumor tissues and paired normal tissues. **(C)** Differential expression of GJA5 in ccRCC in the GEO database. **(D)** Differential expression of GJB1 in ccRCC in the GEO database. **(E)** KM analysis of GJA5 in ccRCC in the TCGA database. **(F)** KM analysis of GJB1 in ccRCC in the TCGA database. **(G)** Analysis of the combined effect of GJA5 and GJB1 in ccRCC in the TCGA database.

The Pan-cancer analysis revealed that GJA5 exhibited the highest expression level in ccRCC, whereas GJB1 was the 12th highest in terms of expression in ccRCC (**Supplementary Figures 2A, B**). Notably, GJA5 and GJB1 were differentially expressed between tumor tissues and matched normal tissues in 15 distinct cancers (**Supplementary Figures 2C, D**), including breast invasive carcinoma, renal chromophobe cell carcinoma, renal clear cell carcinoma, renal papillary cell carcinoma, endometrial carcinoma, thyroid carcinoma, prostate cancer, hepatocellular carcinoma, and lung squamous cell carcinoma. These findings suggested that GJA5 and GJB1 might play crucial roles in the development and progression of urological tumors, as well as a variety of human malignant tumors. These findings warrant further in-depth investigation. Employing the TS dataset, a comprehensive analysis was conducted to examine the differences in gene expression between GJA5 and GJB1 in tumor tissues and normal tissues. The findings demonstrated that both GJA5 and GJB1 exhibited decreased expression in tumor tissues and elevated expression in normal tissues (*p*< 0.001) (**Figures 3A, B**). These findings suggested that the expression of GJA5 and GJB1 was repressed during the occurrence of ccRCC, a phenomenon that was consistent with that observed in additional independent cohorts (GSE73731, GSE95425) (**Figures 3C, D**). Finally, this study further validated the differential expression of GJA5 and GJB1 in ccRCC using the oncopression database (http://www.oncopression.com/downloads.html) (**Supplementary Figure 3**. The results showed that the expression of GJA5 and GJB1 in normal tissue of ccRCC was significantly higher than that in tumoral tissue. Ultimately, the prognostic significance of GJA5 and GJB1 was further assessed. In the TS dataset, KM analysis revealed a significant correlation between low expression of GJA5 and GJB1 and a shorter OS (*p*< 0.05) (**Figures 3E, F**). Patients exhibiting concurrent low expression of GJA5 and GJB1 had the shortest OS, while those with high expression of GJA5 and GJB1 had a prolonged OS (**Figure 3G**). Given the results of the differential expression analysis, we speculated that GJA5 and GJB1 act as tumor suppressors in ccRCC.

#### 3.1.3 Establishment of the RS prognostic model and validation by using clinical characteristics

The RS for each patient was calculated using the following formula:


Risk score =GJA5× (−0.0003) +GJB1× (−0.0005)

**Figure 4:**
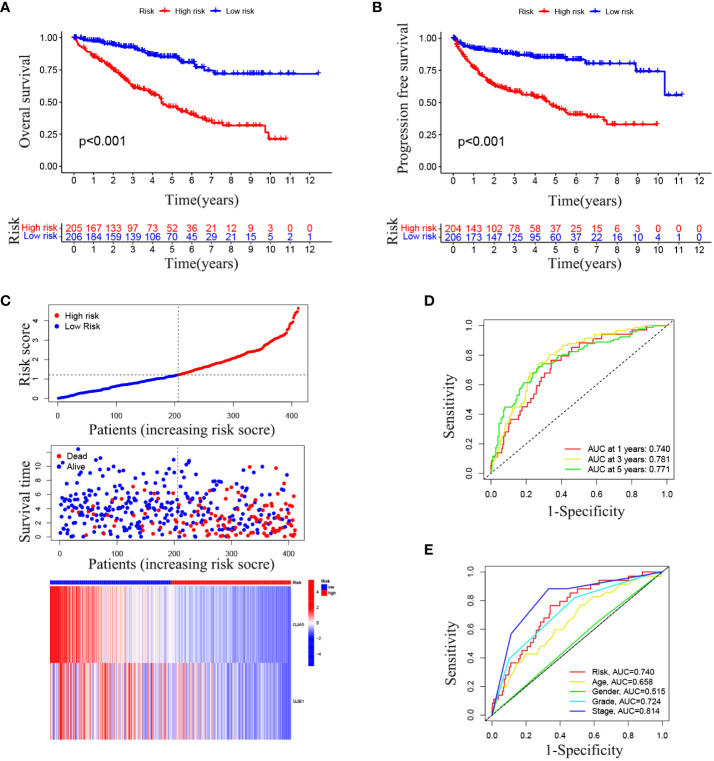
**(A)** OS analysis between the high-RS score group and low-RS score group. **(B)** PFS analysis between the high-RS score group and low-RS score group. **(C)** RS prognosis model for TS (including the RS curve, survival time and survival status of patients, and GRPS gene expression). **(D, E)** ROC curve of the RS prognosis model of TS.

The OS and PFS of patients in the high-RS subgroup were markedly inferior to those in the low-RS subgroup (*p*< 0.001) (**Figures 4A, B**). A prognostic model for RS was developed, encompassing hazard curves, survival scatter plots, and heatmaps of GJA5 and GJB1 expression. The model revealed that patients in the high-RS subgroup exhibited shorter survival, greater mortality, and decreased GJA5 and GJB1 expression (**Figure 4C**). The ROC curve demonstrated that the RS prognostic model exhibited a potent ability to predict patient survival rates (the AUC for predicting 1-year, 2-year, and 3-year survival rates were 0.740, 0.781, and 0.771, respectively). In comparison with other clinical traits, the RS model exhibited strong credibility in predicting patient survival rates (**Figures 4D, E**). Finally, the differences in clinical characteristics between the high-RS and low-RS subgroups were verified. The findings indicated that, in the high-RS subgroup, ccRCC patients exhibited a greater malignancy grade and a greater probability of tumor metastasis, with a greater proportion of male patients than female patients (**Table 2**).

**Table 2:** Differential analysis of clinicopathological parameters between the high-RS subgroup and the low-RS subgroup.

Variable	Sum	High-RS	Low-RS	Chi-square value	*P*
Survival state					
Survival	259(66.24%)	93(47.69%)	166(84.69%)	58.203	< 0.001
Death	132(33.76%)	102(52.31%)	30(15.31%)		
Age					
≤65	258(65.98%)	122(62.56%)	136(69.39%)	1.735	0.188
>65	133(34.02%)	73(37.44%)	60(30.61%)		
Gender					
Female	142(36.32%)	54(27.69%)	88(44.9%)	11.779	< 0.001
Male	249(63.68%)	141(72.31%)	108(55.1%)		
Tumor grade					
G1-2	142(36.32%)	54(27.69%)	88(44.9%)	18.568	< 0.001
G3-4	249(63.68%)	141(72.31%)	108(55.1%)		
Neoplasm staging					
Stage I-II	184(47.06%)	70(35.9%)	114(58.16%)	40.380	< 0.001
Stage III-IV	207(52.94%)	125(64.1%)	82(41.84%)		

#### 3.1.4 Validation of the RS prognostic model

**Figure 5:**
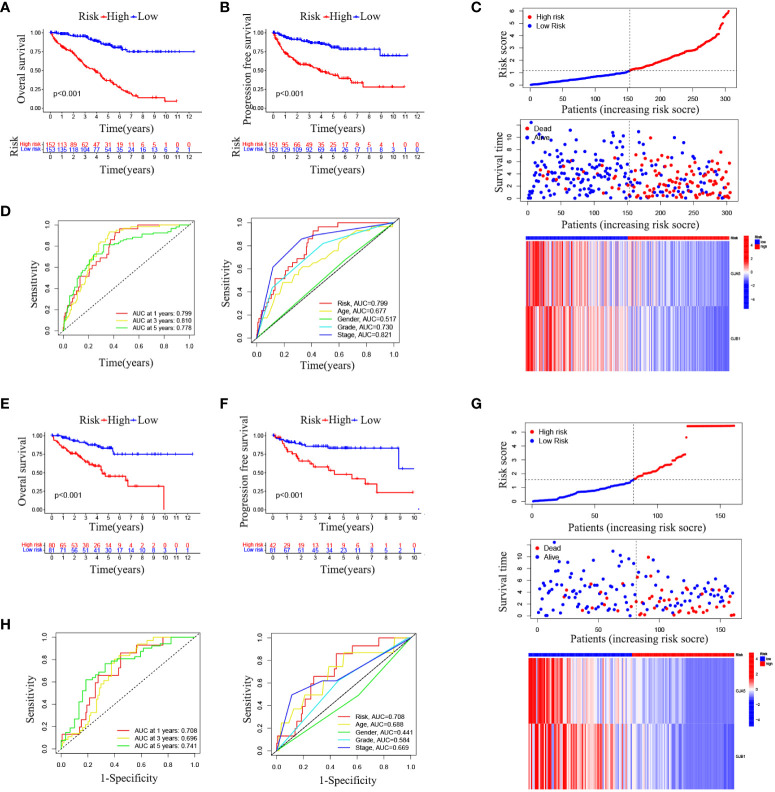
**(A)** OS analysis results in the training set. **(B)** PFS analysis results in the training set. **(C)** RS prognostic model in the training set. **(D)** ROC curve analysis of the RS prognostic model in the training set. **(E)** OS analysis results in the testing set. **(F)** PFS analysis results of testing set. **(G)** RS prognostic model of testing set. **(H)** ROC curve analysis of the RS Prognostic model in the testing Set.

Combined with the GSE29609 dataset, a training set and a testing set of TSs were constructed to assess the prognostic significance of the GRPS in ccRCC. The chi-square test revealed no significant differences in the clinicopathological traits between the training and testing sets (**Table 3**), indicating that the study’s grouping was random and reasonable. In both the training and testing sets, patients in the high-RS subgroup exhibited significantly poorer OS and PFS than did those in the low-RS subgroup (**Figures 5A, B, E, F**). Subsequently, the RS prognostic model was constructed on the training cohort and validated using ROC curve analysis (**Figures 5C, D**). These results align with those for TS, demonstrating that the RS prognostic model in the training cohort was also reliable at predicting the patient survival status (the AUC for predicting 1-year, 3-year, and 5-year survival rates were 0.750, 0.778 and 0.775, respectively). Finally, comparable outcomes were obtained in the testing cohort (**Figure 5G**). The AUC of the RS prognostic model in the testing cohort for predicting 1-year, 3-year, and 5-year survival was 0.708, 0.696, and 0.741, respectively (**Figure 5H**).

**Table 3:** Analysis of differences in clinical traits between training set and testing set n (n%).

Variable	Sum	Training	Testing	Chi-square value	*P*
Survival state					
Survival	281(65.50%)	175(63.64%)	106(68.83%)	0.960	0.327
Death	148(34.50%)	100(36.36%)	48(31.17%)		
Age					
≤65	279(65.03%)	184(66.91%)	95(61.69%)	0.965	0.326
>65	150(34.97%)	91(33.09%)	59(38.31%)		
Tumor grade					
G1-2	197(45.92%)	125(45.45%)	72(46.75%)	0.025	0.875
G3-4	232(54.08%)	150(54.55%)	82(53.25%)		
Neoplasm staging					
Stage I-II	250(58.28%)	165(60.00%)	85(55.19%)	0.750	0.386
Stage III-IV	179(41.72%)	69(44.81%)	110(40.00%)		

#### 3.1.5 Construction and validation of clinical nomograms

To further explore the relationship between the RS prognostic model and clinical characteristics, univariate and multivariate stepwise Cox analysis were performed. The results showed that risk score, age, and tumor stage were closely associated with survival time and clinical outcomes of ccRCC patients, and could serve as independent prognostic factors (**Table 4**). The risk of death for individuals with high-RS was 1.695 times greater than that for those with low-RS (HR = 1.695, 95%CI = 1.439-1.996, *p*< 0.001).

**Table 4:** Univariate and multivariate stepwise Cox regression analysis of RS and clinical characteristics in ccRCC.

	Univariate Cox regression		Multivariable Cox regression	
	*p*-value	Hazard ratio	*p*-value	Hazard ratio
Age	**< 0.001**	1.032(1.017-1.047)	**< 0.001**	1.036(1.018-1.053)
Gender	0.935	0.986(0.692-1.403)	0.994	1.001(0.695-1.444)
Grade	**< 0.001**	2.204(1.752-2.773)	0.162	1.203(0.929-1.558)
Stage	**< 0.001**	1.845(1.591-2.140)	**< 0.001**	1.574(1.327-1.867)
Risk score	**< 0.001**	1.944(1.685-2.244)	**< 0.001**	1.695(1.439-1.996)

The results with bold font indicate statistical significance (*p* < 0.05).

**Figure 6:**
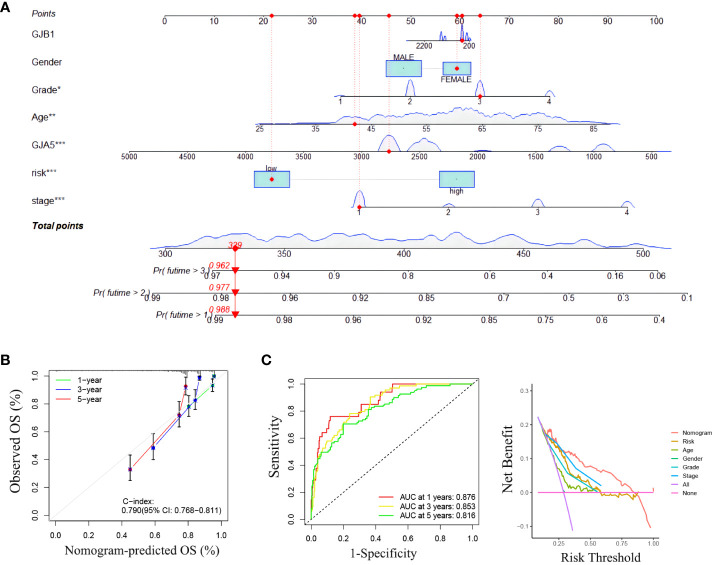
**(A)** Clinical nomogram. **(B)** The calibration curve for predicting 1-year, 3-year and 5-year survival rates with a nomograph. **(C)** ROC curve and DCA curve of the nomogram.

A nomogram incorporating clinicopathological traits (tumor grade, tumor stage), the gene expression pattern of the GRPS, and risk grouping was constructed to assist clinicians in making initial predictions of OS in ccRCC patients (**Figure 6A**). The clinical nomogram was subsequently validated. The calibration curves demonstrated good agreement between the predicted probabilities generated by the nomogram and the actual observed OS values at 1, 2, and 3 years (**Figure 6B**). The results of the ROC curve and DCA analyses revealed that the nomogram exhibited excellent reliability in predicting the survival rate of ccRCC patients (the AUC of the nomogram for predicting the survival rate of patients at 1, 3 and 5 years was 0.876, 0.853 and 0.816, respectively) (**Figure 6C**).

#### 3.1.6 GO and KEGG enrichment analysis

**Figure 7:**
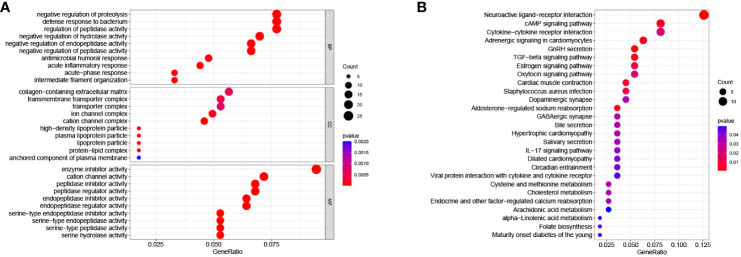
**(A)** GO enrichment analysis. **(B)** KEGG enrichment analysis.

Among the total differential expressed 899 genes, 540 genes were highly expressed in the high-RS subgroup, while the other 359 genes exhibited high expression in the low-RS subgroup (**Supplementary Figure 4**). To determine the biological functions and pathways active in the high-RS subgroup, further exploration was conducted. The results of GO enrichment analysis (**Figure 7A**) revealed that Biological Processes such as “negative regulation of proteolysis”, Cellular Component such as “collagen-containing extracellular matrix”, and Molecular Function such as “enzyme inhibitor activity” were enriched in the high-RS population. Additionally, KEGG enrichment analysis indicated that “neuroactive ligand-receptor interaction”, “cAMP signaling pathway”, “cytokine-cytokine receptor interaction”, “adrenergic signaling pathway in cardiomyocytes”, “GnRH secretion”, and “TGF-beta signaling pathway” were enriched in high-RS population (**Figure 7B**).

#### 3.1.7 Immunocyte infiltration, TME and TMB analysis

**Figure 8:**
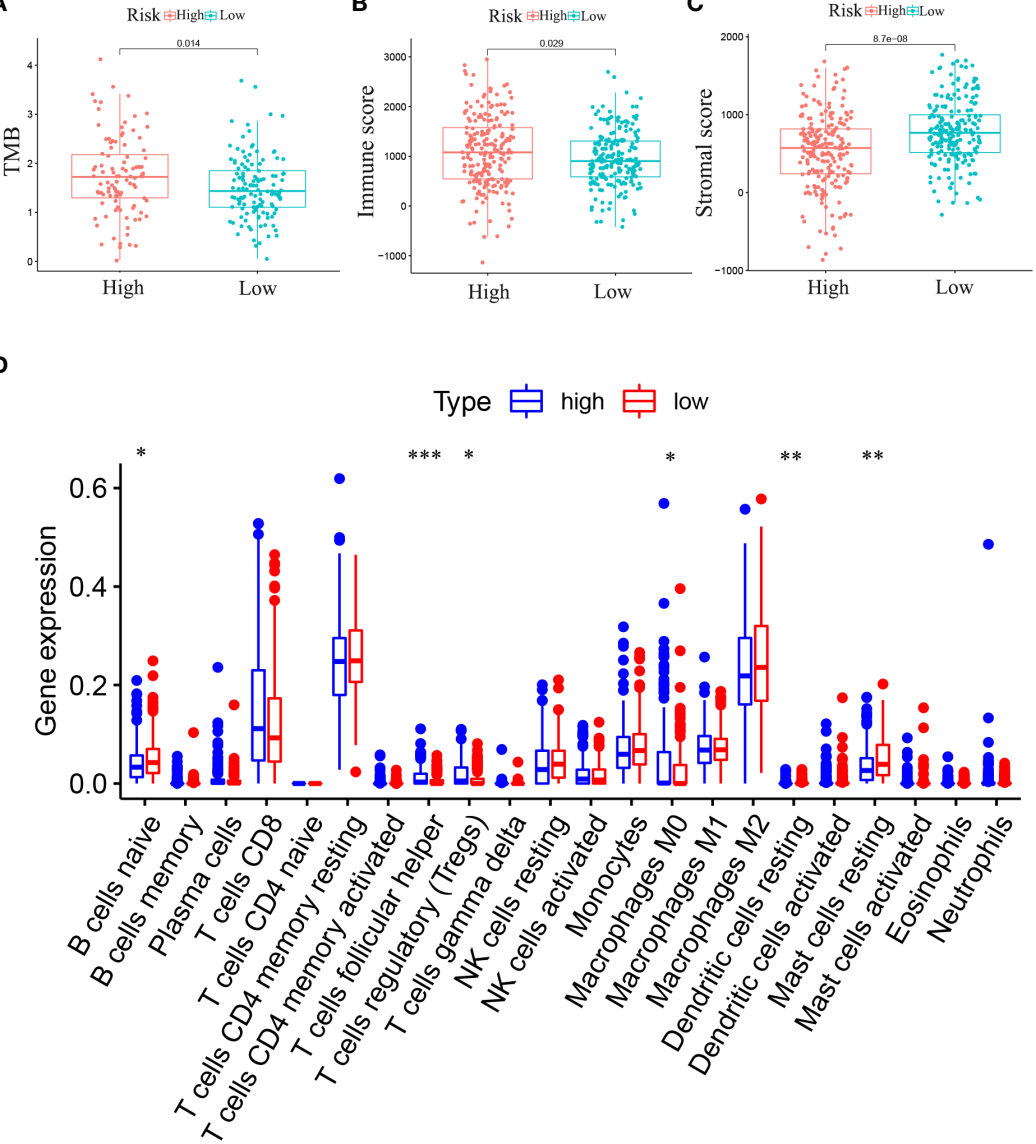
**(A)** Analysis of the difference in TMB between high-RS and low-RS groups. **(B)** Analysis of the Difference in Immune Score between high-RS and low-RS Groups. **(C)** Analysis of the Difference in Stromal Score between high-RS and low-RS Groups. **(D)** Differential expression of immune cells between high-RS and low-RS groups (Immune cell content in the low-RS group is represented in red, and that in the high-RS group is represented in blue. (*p< 0.05, **p< 0.01, ***p< 0.001).

Differences in 22 immune cell types and TMB in the RS group were investigated. Patients in high-RS subgroup presented with higher TMB than that in low-RS subgroup (**Figure 8A**). Compared to those in the low-RS subgroup, the TME in the high-RS subgroup exhibited greater immune cell infiltration and a lower stromal cell content (**Figures 8B, C**). As for TME-related molecular abundance, statistically significant differences were detected for naive B cells (*p<* 0.05), T cell follicular helper cells (*p*< 0.001), regulatory T cells (*p*< 0.05), M0 macrophages (*p*< 0.05), stationary dendritic cells (*p*< 0.01), stationary mast cells (*p*< 0.01), immune cell content (*p*< 0.05) and stromal cell content (*p*< 0.001)(**Figure 8D**). These findings offer a new perspective for exploring the mechanism of individualized immunotherapy in ccRCC patients and the role of GJPs in the occurrence and development of ccRCC. And low expression of GJA5 and GJB1 might be associated with an imbalance in immune homeostasis and compromised responses to immunotherapy in ccRCC patients.

#### 3.1.8 Drug sensitivity analysis

**Figure 9:**
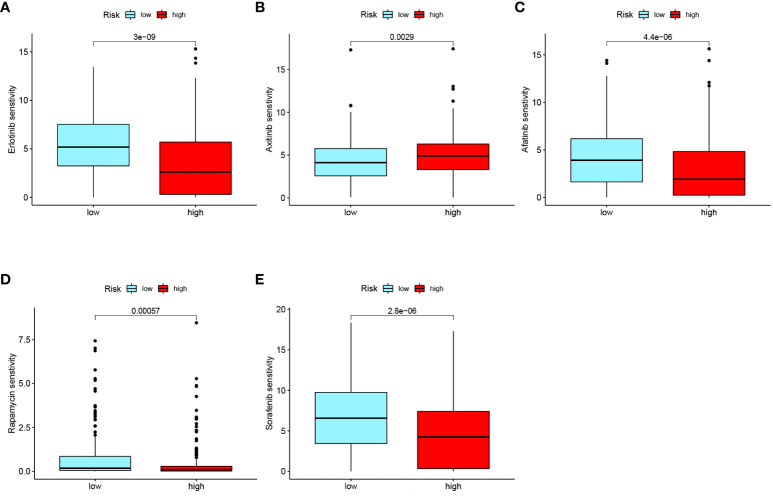
Drug sensitivity analysis of common chemotherapeutic drugs in ccRCC risk groups: **(A)** Erlotinib. **(B)** Axitinib. **(C)** Afatinib. **(D)** Rapamycin. **(E)** Sorafenib.

Drug sensitivity analysis of commonly used chemotherapeutic drugs for ccRCC was conducted based on the RS grouping (**Figures 9A-E**). Among the several chemotherapy drugs used to treat advanced ccRCC, erlotinib (*p<* 0.001), axitinib (*p* = 0.0029), afatinib (*p*< 0.001), rapamycin (*p*< 0.001), and sorafenib (*p<* 0.001) were found to be significantly different among the risk groups. Notably, axitinib exhibited lower sensitivity in the low-RS subgroup, while afatinib, erlotinib, rapamycin, and sorafenib had lower sensitivity in the high-RS subgroup. These findings offer new insights into individualized targeted drug therapy for patients with advanced ccRCC.

### 3.2 Cytological experimental verification of GJA5 and GJB1 results

#### 3.2.1 Results of GJA5 and GJB1 expression in renal cell lines

**Figure 10:**
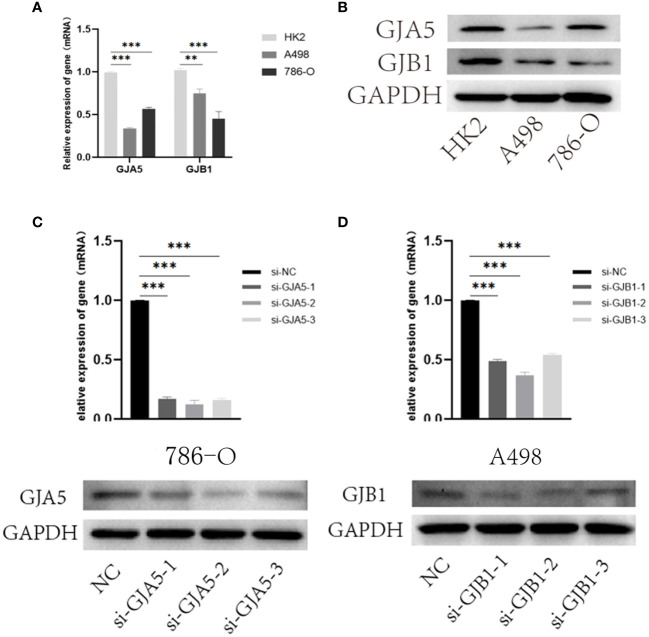
**(A)** qRT-PCR results of GJA5 and GJB1 expression at the RNA level. **(B)** Western blot results of GJA5 and GJB1 expression at protein level. **(C)** qRT-PCR and Western blot results of GJA5 in 786-O cells after transfection with three small interfering RNAs. **(D)** qRT-PCR and Western blot results of GJB1 in A498 cells after transfection with three small interfering RNAs. (*p< 0.05, **p< 0.01, ***p< 0.001).

Furthermore, the expression of GJA5 and GJB1 at the protein level was detected via Western blot analysis, and the expression of GJA5 and GJB1 at the RNA level was detected via qRT-PCR. qRT-PCR revealed that the mRNA levels of GJA5 and GJB1 in renal tumor cells (A498 and 786-O) were lower than those in noncancerous renal cells (HK2) (**Figure 10A**) and this pattern was confirmed at the protein level (**Figure 10B**). Moreover, according to the qRT-PCR and Western blot detection results, at both the RNA and protein expression levels, GJB1 was expressed at higher levels in A498 cells, while GJA5 was expressed at higher levels in 786-O cells. Therefore, functional experiments were conducted to knock down GJB1 in the A498 cell line and GJA5 in the 786-O cell line.

#### 3.2.2 Construction of GJA5 and GJB1 knockdown cell lines

Furthermore, to investigate the effects of GJA5 and GJB1 interference on the cytological behavior of ccRCC cells, at least three independent cell experiments were conducted (**Figures 10C, D**). The expression of GJA5 in the 786-O cell line and GJB1 in the A498 cell line were specifically targeted and knocked down by chemically synthesized siRNA. Western blot analysis revealed that si-GJA5-2, si-GJA5-3, si-GJB1-1 and si-GJB1-2 had relatively high knockdown efficiencies. Therefore, the above four kinds of siRNAs were selected for subsequent cell behavioral experiments.

#### 3.2.3 Low expression of GJA5 and GJB1 promote the proliferation, migration and EMT and inhibit apoptosis of ccRCC cells

**Figure 11:**
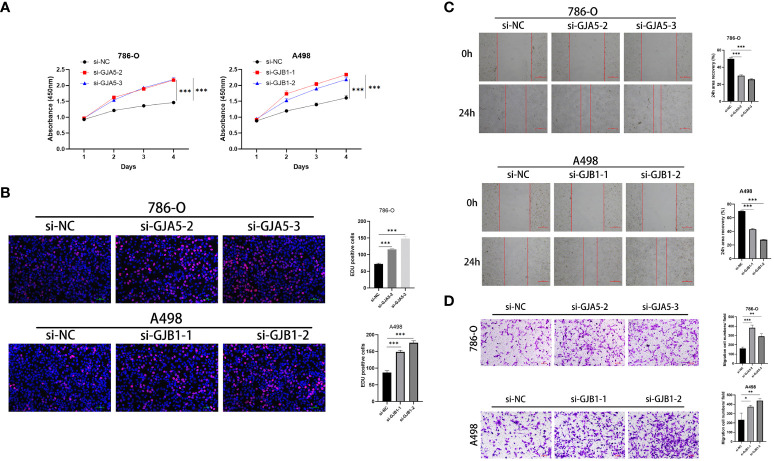
**(A)** The CCK8 experiment results of GJA5 and GJB1 knockdown on the cell proliferation in 786-O cells and A498 cells, respectively. **(B)** The EdU experiment results of GJA5 and GJB1 knockdown on the cell proliferation in 786-O cells and A498 cells, respectively (Error bar = 50 μm). **(C)** The cell scratch test results of GJA5 and GJB1 knockdown on the cell migration in 786-O cells and A498 cells, respectively (Error bar = 500 μm). **(D)** The Transwell experiment results of GJA5 and GJB1 knockdown on the cell migration potential in 786-O cells and A498 cells, respectively (Error bar = 100 μm). (*p < 0.05, **p < 0.001, ***p < 0.01).

**Figure 12:**
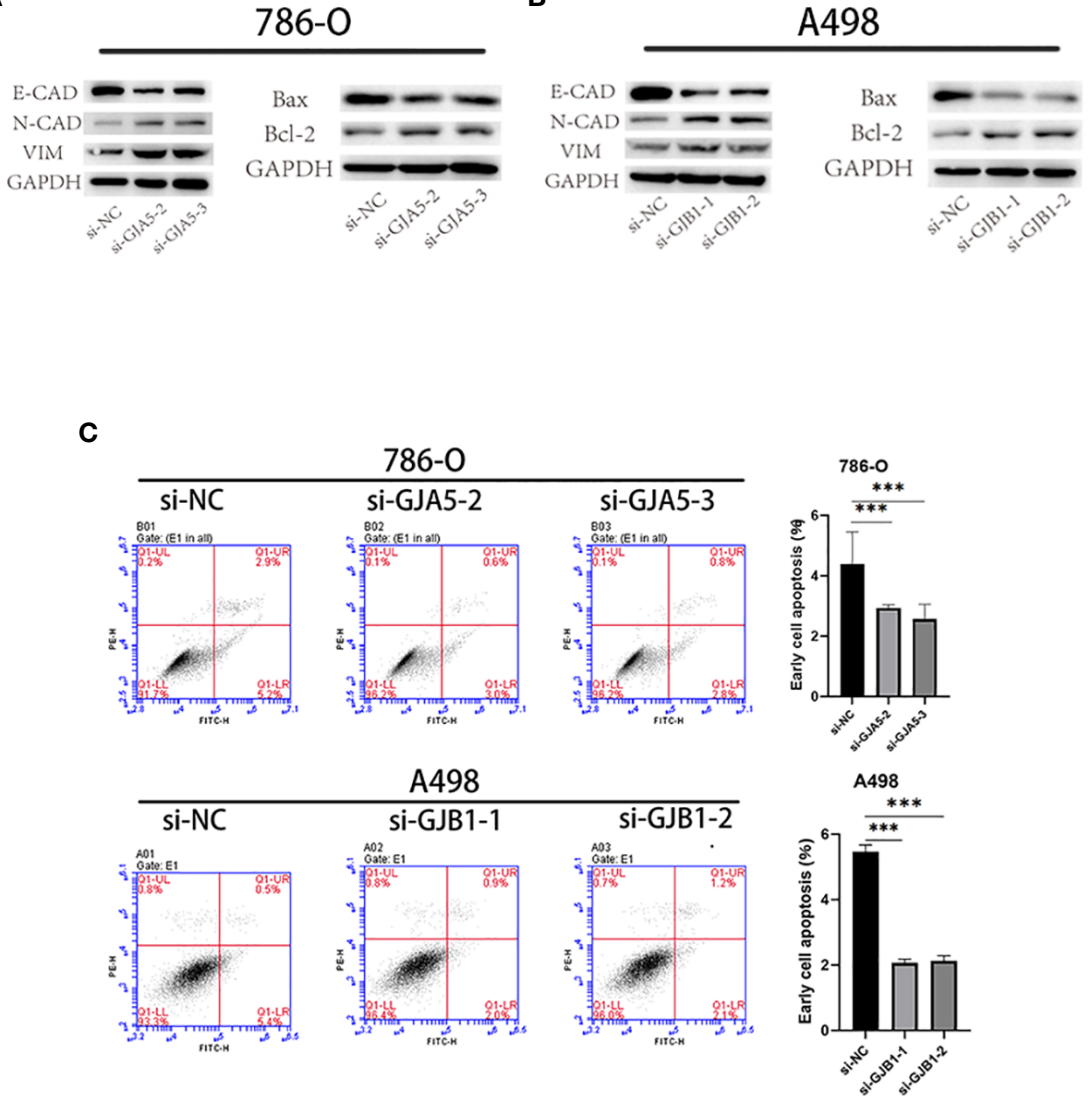
**(A)** After knocking down GJA5, the protein expression of E-cad, N-cad, VIM, Bax, and Bcl-2 was detected in the 786-O cell line. **(B)** After knocking down GJB1, the protein expression of E-cad, N-cad, VIM, Bax, and Bcl-2 was detected in the A-498 cell line. **(C)** Flow cytometry analysis of cell apoptosis after knockdown of GJA5 and GJB1 genes in 786-O and A498 cells, respectively. (*p < 0.05, **p < 0.001, ***p < 0.01).

CCK-8 and EdU assays demonstrated that cell viability was sharply increased in the si-GJA5 and si-GJB1 groups (**Figures 11A, B**). Similarly, transwell and wound healing assays demonstrated that cell migration increased significantly after GJA5 and GJB1 were knocked down (**Figures 11C, D**). Western blot analysis revealed that after GJA5 or GJB1 knockdown, the expression of E-cad and Bax in ccRCC cells decreased, while the expression of N-cad, VIM and Bcl-2 increased (**Figures 12A, B**). Flow cytometry showed that knockdown of GJA5 or GJB1 inhibited cells apoptosis (**Figure 12C**). Collectively, these results indicate that GJA5 and GJB1 may inhibit ccRCC development.

## 4 Discussion

Gap junctions are intercellular channel clusters formed on the plasma membrane by the connexin family of proteins expressed by GJPs (21, 22), which allows the diffusion of ions or small molecules and the transmission of electrical signals (23, 24). As important channels of intercellular communication, gap junction proteins provide a basis for the cooperative work and functional integrity of various systems and organs in organisms (25, 26). Genetic or acquired changes in connexin proteins are closely related to cancer (27). Abnormal GJP expression is related to the recurrence and metastasis of cancer and to increase patient mortality. This difference may be due to the loss of channel function, which leads to a decrease in tumor inhibition ability (usually called tumor dormancy) (28, 29), and this inhibition ability depends on the function of gap junction coupling among cells (30). Moreover, when the gap junction channel is damaged, the permeability and sensitivity to chemotherapy drugs are reduced (31, 32). For example, defects in GJA1 function mediate the resistance of breast cancer to tamoxifen (33). Destruction of GJB1 plays a role in promoting the occurrence and development of ovarian cancer and is not conducive to the action of chemical drugs (34). Mutations and loss of function of the GJA5 gene are closely related to the occurrence of isolated ventricular fibrillation in humans (35). Therefore, GJPs are crucial to the steady state of the human body. However, the role of GJPs in ccRCC is unclear. In our study, we comprehensively analyzed the biological function, pathway, clinical significance and prognostic value of GJPs in patients with ccRCC, which filled the gap in the study of GJPs in ccRCC.

In addition, the occurrence and development of cancer are always accompanied by changes in the tumor microenvironment and the triggering of immune escape mechanisms (36, 37). When gap junctions are blocked, humoral and CD8+ T-cell immune responses are inhibited or even eliminated (38). It has been proven that there are many gap junction channels between immune cells, such as GJA5 in T and B lymphocytes (39), and GJB1 in mast cells (40), and that communication between cardiac cells is promoted by cardiac macrophages through gap junctions (41). Therefore, the stability of GJP function is crucial for the immune system to function. However, the role of GJPs in ccRCC remains unclear. We analyzed the clinical significance and biological function of GJPs in ccRCC through a public database system. Furthermore, we developed the GRPS, a novel tumor-related prognostic feature. Low expression levels of GJA5 and GJB1 predict poor prognosis in ccRCC patients, and could be used as independent prognostic markers and drug therapeutic targets for ccRCC. Additionally, GJA5 and GJB1 were verified by cellular functional experiments, and the results showed that GJA5 and GJB1 could be used as prognostic markers for ccRCC.

In this study, LASSO and Cox regression models were developed for 21 GJPs to identify genes with independent prognostic effects, and the relationships between GRPS and tumor grade, tumor stage and survival status of patients were further explored. The results showed that GJA5 and GJB1 have independent prognostic value in ccRCC. The RS was subsequently calculated for each patient, and an RS prognostic model was constructed. Analysis of the ROC curve confirmed that the prediction model developed in this study is reliable, indicating its potential application in clinical practice for predicting the prognosis of patients with ccRCC.

Moreover, a clinical nomogram that incorporates the GPRS score, patient clinical characteristics, and the RS was developed. ROC curve and DCA analyses demonstrated that the nomogram is highly sensitive for predicting the survival status of patients (AUC values for 1-, 3-, and 5-year were 0.876, 0.853 and 0.816, respectively). To assess the generalizability of the RS prognostic model, this study combined it with external datasets, and the results were positive. Further investigations revealed correlations between the GRPS and immune cell infiltration, the TME, and the TMB. Drug sensitivity analysis indicated that the high-RS subgroup was more sensitive to afatinib, erlotinib, rapamycin, and sorafenib, while the low-RS subgroup exhibited greater sensitivity to axitinib, which is conducive to the development of individualized chemotherapy regimens for patients with advanced ccRCC.

This study has certain limitations. Firstly, the dataset examined in this analysis was merely derived from the public (TCGA and GEO) database, and has a limited sample size. The findings still need to be further confirmed by a multi-database and multi-center study. Secondly, although our constructed model obtained promising results, further clinical experiments are necessary to validate and assess the efficacy of our model.

In conclusion, this study established a more economical and personalized prognostic model that is easy to apply and could provide clinicians with new ideas for the prognosis and treatment of ccRCC patients.

## Data availability statement

Publicly available datasets were analyzed in this study. This data can be found here: TCGA database (https://portal.gdc.cancer.gov/) and GEO database (https://www.ncbi.nlm.nih.gov/geo/), Microarray data (GSE29609, GSE95425, GSE73731).

## Author contributions

YH: Conceptualization, Data curation, Software, Visualization, Writing – original draft. WG: Formal analysis, Validation, Visualization, Writing – original draft. YuZ: Conceptualization, Investigation, Software, Writing – review & editing. XW: Conceptualization, Writing – review & editing. BF: Conceptualization, Writing – review & editing. YiZ: Conceptualization, Writing – review & editing. LY: Project administration, Supervision, Writing – review & editing. ZL: Funding acquisition, Methodology, Software, Supervision, Writing – review & editing. GG: Conceptualization, Funding acquisition, Investigation, Resources, Writing – review & editing.

## Supplementary material

Supplementary Table 1 General clinical data of ccRCC patients involved in data analysis in this study.

Supplementary Table 2 Univariate Cox regression results for gap junction protein gene.

Supplementary Figure 1 The Kaplan-Meier survival analysis for 5 GJPs with differential survival significance: **(A)** GJA1 **(B)** GJA4 **(C)** GJA5 **(D)** GJB1 **(E)** GJC1.

Supplementary Figure 2 GJA5 and GJB1 expression and differential expression in pan-cancer: **(A)** Expression of GJA5 in pan-cancer **(B)** Expression of GJB1 in pan-cancer **(C)** Differential expression of GJA5 between normal and tumoral tissues in pan-cancer **(D)** Differential expression of GJB1 between normal and tumoral tissues in pan-cancer.

Supplementary Figure 3 In the oncopression database, the differential expression of GJA5 and GJB1 in ccRCC tumor tissue and normal tissue: **(A)** Differential expression of GJA5 between normal and tumoral kidney tissues in the Oncopression database **(B)** Differential expression of GJB1 between normal and tumoral kidney tissues in the Oncopression database.

Supplementary Figure 4 Visualization of differentially expressed genes (*P*< 0.05, logFC = 1, FDR< 1) in risk groups: **(A)** Heatmap **(B)** Volcano plot.
