# Supplementary material for: Identification and validation of a gap junction protein related signature for predicting the prognosis of renal clear cell carcinoma
Source: Front Oncol. 2024 Feb 22;14:1354049. doi: 10.3389/fonc.2024.1354049 (PMC10919056; doi:10.3389/fonc.2024.1354049)
Supplement: Supplementary Table 1 — General clinical data of ccRCC patients involved in data analysis in this study. [file Table_1.docx]

Supplementary Table 1 General clinical data of ccRCC patients involved in data analysis in this study.

|  | TCGA | GSE29609 | GSE95425 | GSE73731 |
| --- | --- | --- | --- | --- |
| Sort |  |  |  |  |
| Normal | 63 |  | 53 |  |
| Tumor | 411 | 38 |  | 256 |
| Age |  |  |  |  |
| ≤65 | 271 | 21 |  |  |
| ＞65 | 140 | 17 |  |  |
| Gender |  |  |  |  |
| Male | 258 |  |  |  |
| Female | 153 |  |  |  |
| Grade |  |  |  |  |
| G1 | 12 | 1 |  |  |
| G2 | 186 | 12 |  |  |
| G3 | 161 | 10 |  |  |
| G4 | 52 | 15 |  |  |
| Stage |  |  |  |  |
| Stage I | 213 | 10 |  |  |
| Stage II | 41 | 3 |  |  |
| Stage III | 95 | 11 |  |  |
| Stage IV | 62 | 14 |  |  |
