# Supplementary material for: Identification and validation of a gap junction protein related signature for predicting the prognosis of renal clear cell carcinoma
Source: Front Oncol. 2024 Feb 22;14:1354049. doi: 10.3389/fonc.2024.1354049 (PMC10919056; doi:10.3389/fonc.2024.1354049)
Supplement: Supplementary Table 2 — Univariate Cox regression results for gap junction protein gene. [file Table_2.docx]

Supplementary Table 2 Univariate Cox regression results for gap junction protein gene.

| Gene | HR | 95% CI | *p* |
| --- | --- | --- | --- |
| GJA1 | 1.000 | 1.000~1.000 | **<0.001** |
| GJA3 | 1.001 | 0.996~1.006 | 0.707 |
| GJA4 | 1.000 | 1.000~1.000 | **<0.001** |
| GJA5 | 0.999 | 0.999~1.000 | **<0.001** |
| GJA8 | 1.006 | 0.979~1.033 | 0.686 |
| GJA9 | 1.011 | 0.942~1.086 | 0.753 |
| GJA10 | 1.043 | 0.928~1.171 | 0.483 |
| GJB1 | 0.999 | 0.999~0.999 | **<0.001** |
| GJB2 | 1.000 | 1.000~1.000 | 0.257 |
| GJB3 | 1.001 | 1.000~1.003 | 0.034 |
| GJB4 | 1.001 | 0.999~1.003 | 0.175 |
| GJB5 | 1.001 | 1.000~1.002 | 0.126 |
| GJB6 | 1.002 | 0.999~1.004 | 0.126 |
| GJB7 | 1.016 | 0.971~1.063 | 0.488 |
| GJC1 | 1.000 | 1.000~1.000 | **<0.001** |
| GJC2 | 1.000 | 0.999~1.001 | 0.869 |
| GJC3 | 0.988 | 0.971~1.005 | 0.168 |
| GJD2 | 1.010 | 0.991~1.031 | 0.303 |
| GJD3 | 1.016 | 0.979~1.053 | 0.406 |
| GJD4 | 1.074 | 1.026~1.125 | 0.002 |
| GJE1 | 0.641 | 0.330~1.243 | 0.188 |
