# Supplementary figures and images for: Identification and validation of a gap junction protein related signature for predicting the prognosis of renal clear cell carcinoma
Source: Front Oncol. 2024 Feb 22;14:1354049. doi: 10.3389/fonc.2024.1354049 (PMC10919056; doi:10.3389/fonc.2024.1354049)

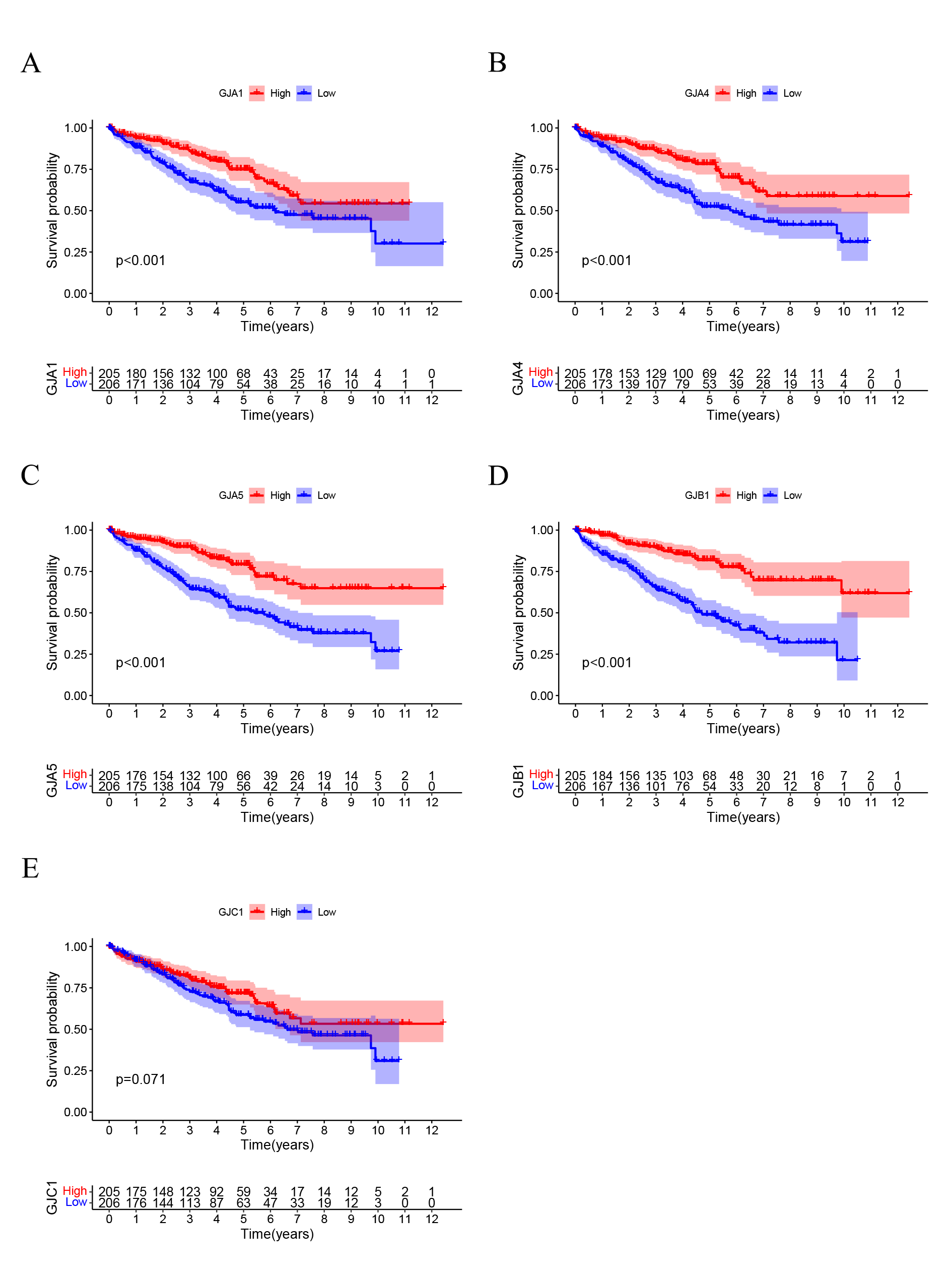

Supplement: Supplementary Figure 1 — The Kaplan-Meier survival analysis for 5 GJPs with differential survival significance: (A) GJA1 (B) GJA4 (C) GJA5 (D) GJB1 (E) GJC1. [file Image_1.tif]

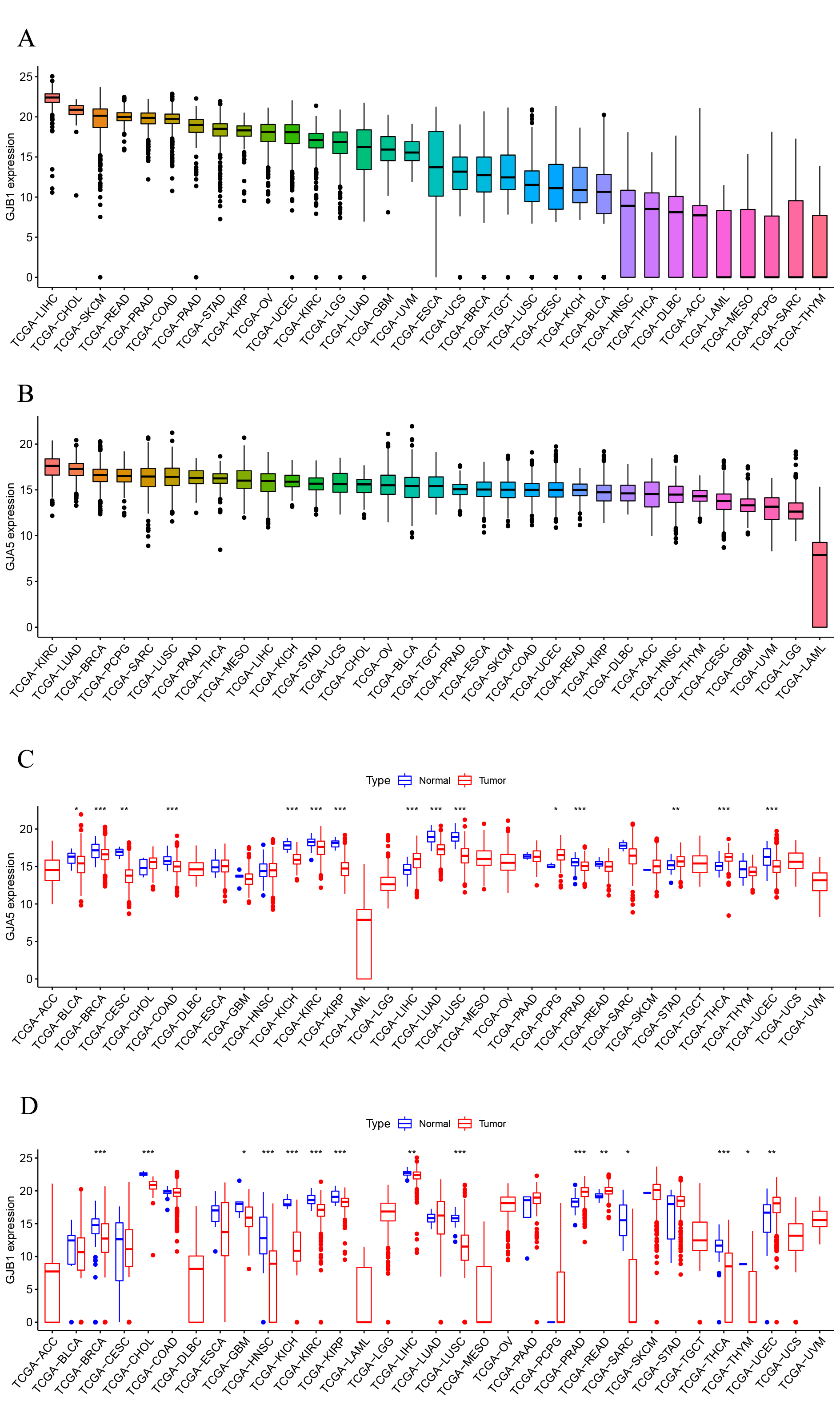

Supplement: Supplementary Figure 2 — GJA5 and GJB1 expression and differential expression in pan-cancer: (A) Expression of GJA5 in pan-cancer (B) Expression of GJB1 in pan-cancer (C) Differential expression of GJA5 between normal and tumoral tissues in pan-cancer (D) Differential expression of GJB1 between normal and tumoral tissues in pan-cancer. [file Image_2.tif]

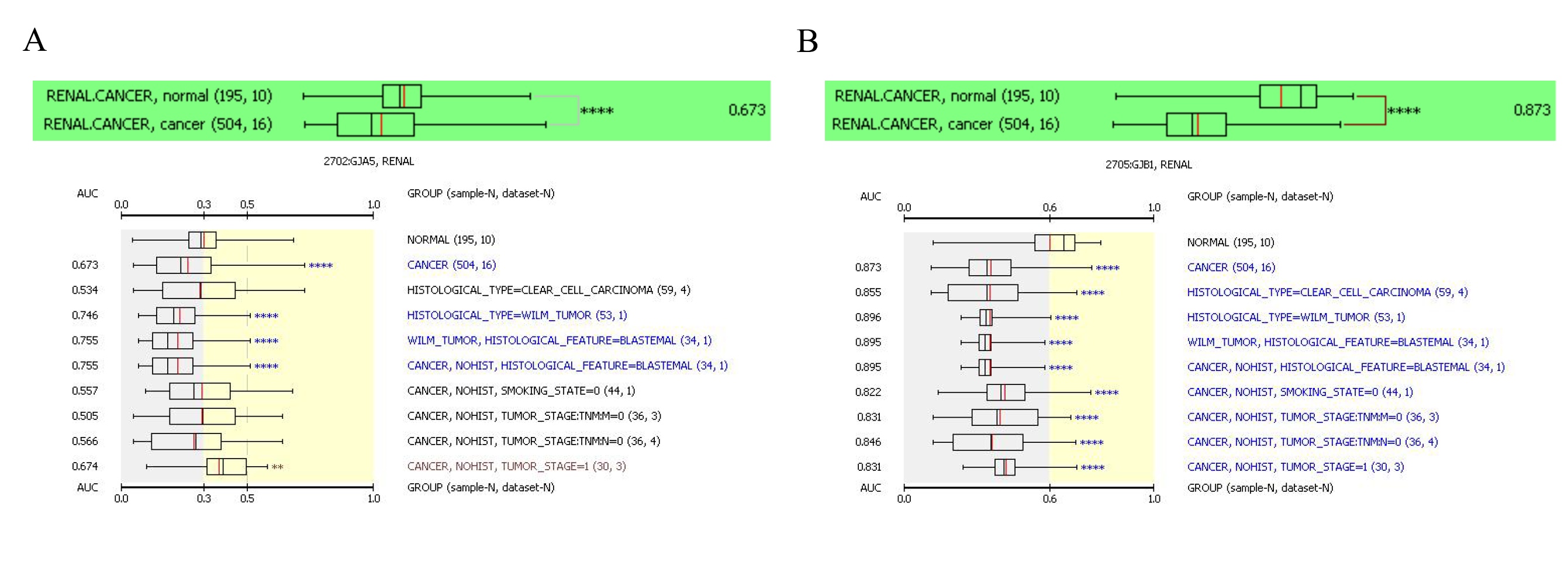

Supplement: Supplementary Figure 3 — In the oncopression database, the differential expression of GJA5 and GJB1 in ccRCC tumor tissue and normal tissue: (A) Differential expression of GJA5 between normal and tumoral kidney tissues in the Oncopression database (B) Differential expression of GJB1 between normal and tumoral kidney tissues in the Oncopression database. [file Image_3.tif]

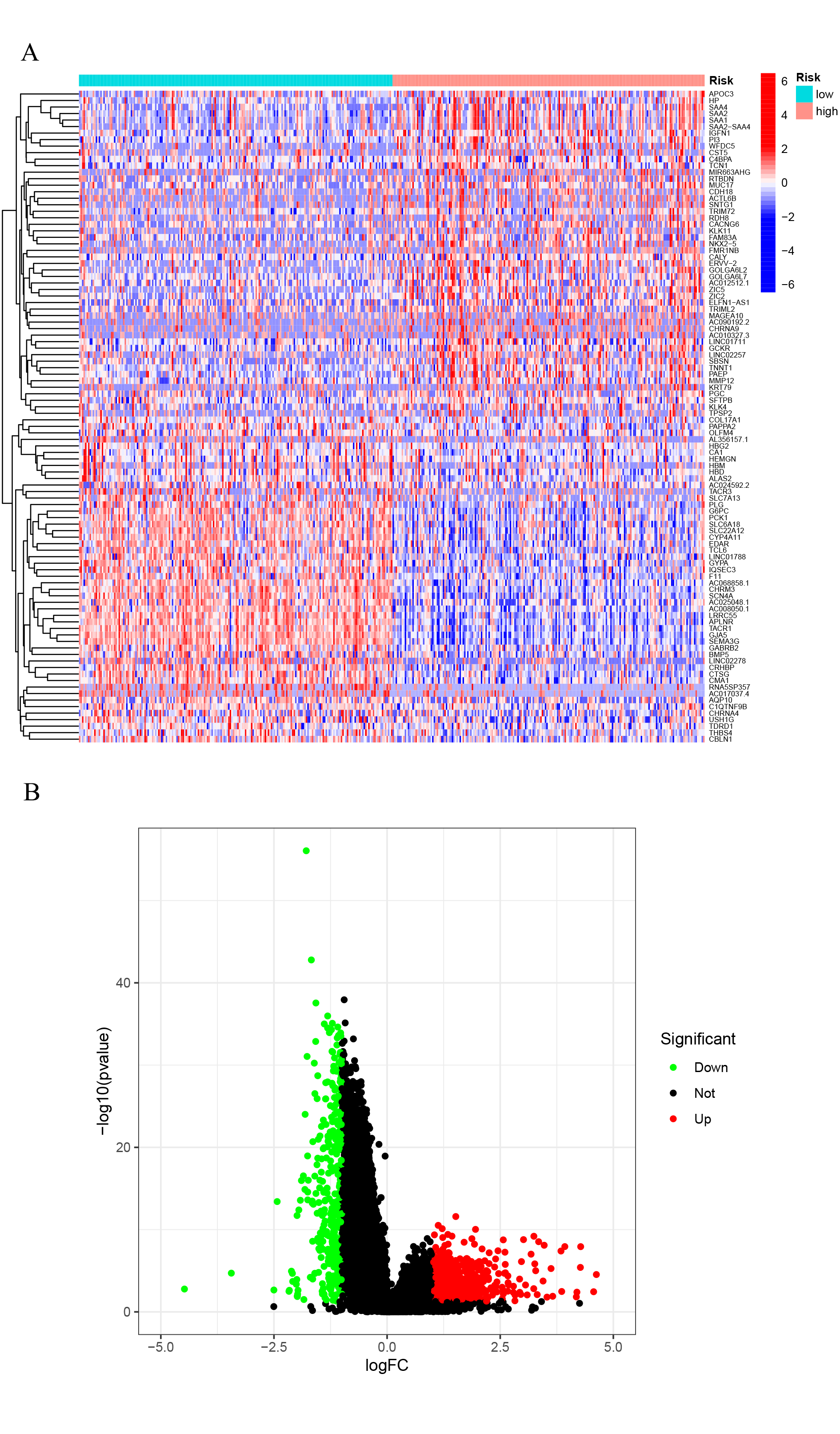

Supplement: Supplementary Figure 4 — Visualization of differentially expressed genes (P< 0.05, logFC = 1, FDR< 1) in risk groups: (A) Heatmap (B) Volcano plot. [file Image_4.tif]
